# Feasibility of Ipsilateral Revision Oblique Lateral Interbody Fusion for Adjacent Segment Disease After Primary Oblique Lateral Interbody Fusion: A Retrospective Case Series

**DOI:** 10.7759/cureus.87961

**Published:** 2025-07-15

**Authors:** Ryuto Tsuchiya, Yasuhiro Shiga, Sumihisa Orita, Yawara Eguchi, Kazuhide Inage, Seiji Ohtori

**Affiliations:** 1 Department of Orthopedic Surgery, Graduate School of Medicine, Chiba University, Chiba, JPN; 2 Center for Frontier Medical Engineering, Chiba University, Chiba, JPN

**Keywords:** adjacent segment disease, lumbar spinal canal stenosis, lumbar spine, oblique lateral interbody fusion, revision surgery

## Abstract

Background: Oblique lateral interbody fusion (OLIF) is a minimally invasive procedure widely used to treat various lumbar spinal disorders. Although OLIF is an effective salvage procedure for adjacent segment disease (ASD) after transforaminal lumbar interbody fusion (TLIF) or posterior lumbar interbody fusion (PLIF), the feasibility of revision OLIF (re-OLIF) for ASD after primary OLIF remains unclear. We aimed to evaluate the feasibility and outcomes of re-OLIF in patients with ASD after primary OLIF.

Materials and methods: This retrospective case series included patients who underwent OLIF at our hospital between 2012 and 2023, developed ASD postoperatively, and subsequently underwent re-OLIF with at least 12 months of follow-up. Clinical data, including operative details, intraoperative blood loss, perioperative complications, and clinical outcomes such as visual analog scale (VAS) and Japanese Orthopaedic Association (JOA) scores, were collected and analyzed.

Results: Six patients underwent re-OLIF for ASD following primary OLIF. Re-OLIF was successfully performed in all cases with no significant complications or difficulties. Operative time and intraoperative blood loss were comparable between the re-OLIF and primary OLIF groups. Clinical outcomes improved significantly after re-OLIF, with VAS scores decreasing from a median of 7.5 to 2.0 and JOA scores increasing from a median of 15.5 to 21.0.

Conclusions: Re-OLIF can be a safe and effective salvage procedure for ASD after primary OLIF, with outcomes comparable to those of primary OLIF. Given the increasing number of OLIF procedures performed, further studies with larger sample sizes are necessary to confirm the long-term safety and efficacy of re-OLIF as a salvage option for ASD.

## Introduction

Oblique lateral interbody fusion (OLIF) is a minimally invasive interbody fusion technique performed via the retroperitoneal approach [1]. Compared to conventional posterior approaches such as transforaminal lumbar interbody fusion (TLIF) and posterior lumbar interbody fusion (PLIF), OLIF allows for the insertion of a larger interbody cage, enabling the restoration of disc height and indirect decompression through ligamentotaxis [2,3]. Due to its minimally invasive nature and favorable clinical outcomes, OLIF has become widely indicated for various lumbar spinal disorders, including lumbar spinal canal stenosis and spondylolisthesis [3,4].

Owing to the use of a retroperitoneal corridor that avoids the posterior surgical field, OLIF has been reported to be an effective salvage option for adjacent segment disease (ASD) after TLIF or PLIF [4-10]. In addition, OLIF is reportedly a useful salvage procedure for recurrent stenosis following decompression surgery [11,12]. However, because OLIF itself is also a form of interbody fusion, it carries a risk of ASD, similar to TLIF and PLIF. Although the exact incidence of ASD following OLIF remains unclear, finite element analysis studies have suggested that OLIF increases mechanical stress on adjacent segments, potentially elevating the risk of ASD [13,14].

Cases of ASD following OLIF are not uncommon; however, there are no comprehensive reports on how best to manage such cases. Although OLIF has been used as a salvage procedure for ASD occurring after TLIF or PLIF, it remains unclear whether revision OLIF (re-OLIF) can also be appropriately performed as a salvage procedure for ASD that develops after primary OLIF. Unlike cases where OLIF is used after posterior surgery, both the initial and revision surgeries in these cases involve the same retroperitoneal corridor, which may lead to adhesions or other technical difficulties. Therefore, this study aimed to investigate the feasibility of performing re-OLIF in patients who developed ASD after primary OLIF and evaluate its clinical outcomes.

## Materials and methods

Study design and setting

The study was conducted at Chiba University Hospital, Japan. Patients were selected according to the following criteria: undergoing OLIF at our hospital between 2012 and 2023, subsequently developing symptomatic ASD, and undergoing an attempted re-OLIF procedure, with a minimum of 12 months of follow-up after re-OLIF.

In this study, symptomatic ASD was defined, based on previously reported criteria [15], as new-onset radiographic degeneration (e.g., disc herniation, spinal canal stenosis, or degenerative spondylolisthesis) at a spinal level adjacent to the prior fusion, accompanied by corresponding clinical symptoms such as low back pain, radiculopathy, or neurogenic claudication. All patients had symptoms refractory to conservative treatments for at least three months and ultimately required surgical intervention. Patients with high-grade spondylolisthesis (Meyerding grade ≥ III [16]), traumatic lesions, infection, or tumors were excluded.

The retrospective case series was conducted using anonymized clinical data, and in accordance with institutional policies, the requirement for institutional review board (IRB) approval was waived. All patients were informed that their clinical data would be used for publication, and written informed consent was obtained.

Surgical procedure

OLIF was performed as described previously [17]. Briefly, the patient was placed in the right lateral decubitus position. After fine positioning adjustment, the target intervertebral level was identified under fluoroscopic guidance. A 4-cm skin incision was made 4-10 cm anterior to the anterior edge of the target disc space. The external oblique, internal oblique, and transversus abdominis muscles are split to enter the retroperitoneal space. Using the index finger, the retroperitoneal space was bluntly separated along the internal abdominal wall toward the quadratus lumborum muscle. The finger was then turned medially to palpate the transverse process, and the dissection was extended toward the psoas major muscle to reach its anterior border. A discectomy and interbody cage placement were performed. In re-OLIF cases, the retroperitoneal approach was similarly performed on the left side, and the target disc space was accessed for cage insertion. After completion of the anterior procedure, the patient was repositioned, prone and posterior fixation was performed using pedicle screws. In re-OLIF cases, pedicle screws at the levels where solid interbody fusion was achieved from the primary OLIF were removed as needed. Direct decompression via the posterior approach was also performed when indicated based on the case.

Clinical measures

The following clinical data were collected: age at the time of re-OLIF, sex, body mass index (BMI) at re-OLIF, history of prior abdominal surgery, spinal level at which re-OLIF was attempted, level at which primary OLIF was performed, and the interval between primary OLIF and re-OLIF. Surgical data included the feasibility of re-OLIF, operative time (total, anterior, and posterior surgical time) for both primary and re-OLIF, intraoperative blood loss during both procedures, and perioperative complications. Changes in visual analogue scale (VAS) and Japanese Orthopaedic Association (JOA) scores [18] before and after re-OLIF were also evaluated.

## Results

Patient characteristics

The patient characteristics are summarized in Table 1. The median age at the time of re-OLIF was 65.5 years (interquartile range (IQR), 47.3-76.8), and the median BMI was 27.2 (IQR, 22.6-28.6). A history of prior abdominal surgery was noted in two of the six patients. The levels at which re-OLIF was attempted included L1/2 in one case, L2/3 in two cases, L3/4 in one case, and L4/5 in two cases. The median interval between the primary OLIF and re-OLIF was 44.6 months (IQR, 31.2-69.1).

**Table 1:** Summary of patient characteristics. BMI: body mass index; OLIF: oblique lateral interbody fusion; re-OLIF: revision OLIF; VAS: visual analogue scale; JOA: Japanese Orthopaedic Association

Case No.	Age	Sex	BMI	History of abdominal surgery	Level of re-OLIF	Level of primary OLIF	Interval to re-OLIF (months)	VAS score before re-OLIF	JOA score before re-OLIF
1	49	M	31.2	No	L3/4	L4/5	74.4	7	15
2	76	F	27.0	Yes	L2/3	L3/4	24.1	8	16
3	79	F	24.2	No	L2/3	L3/4	67.4	9	15
4	42	M	27.8	No	L4/5	L2/3, 3/4	52.2	4	18
5	73	M	17.9	Yes	L4/5	L3/4	36.9	7	16
6	58	M	27.5	No	L1/2	L2/3	33.6	8	10

Clinical outcomes

**Figure 1:**
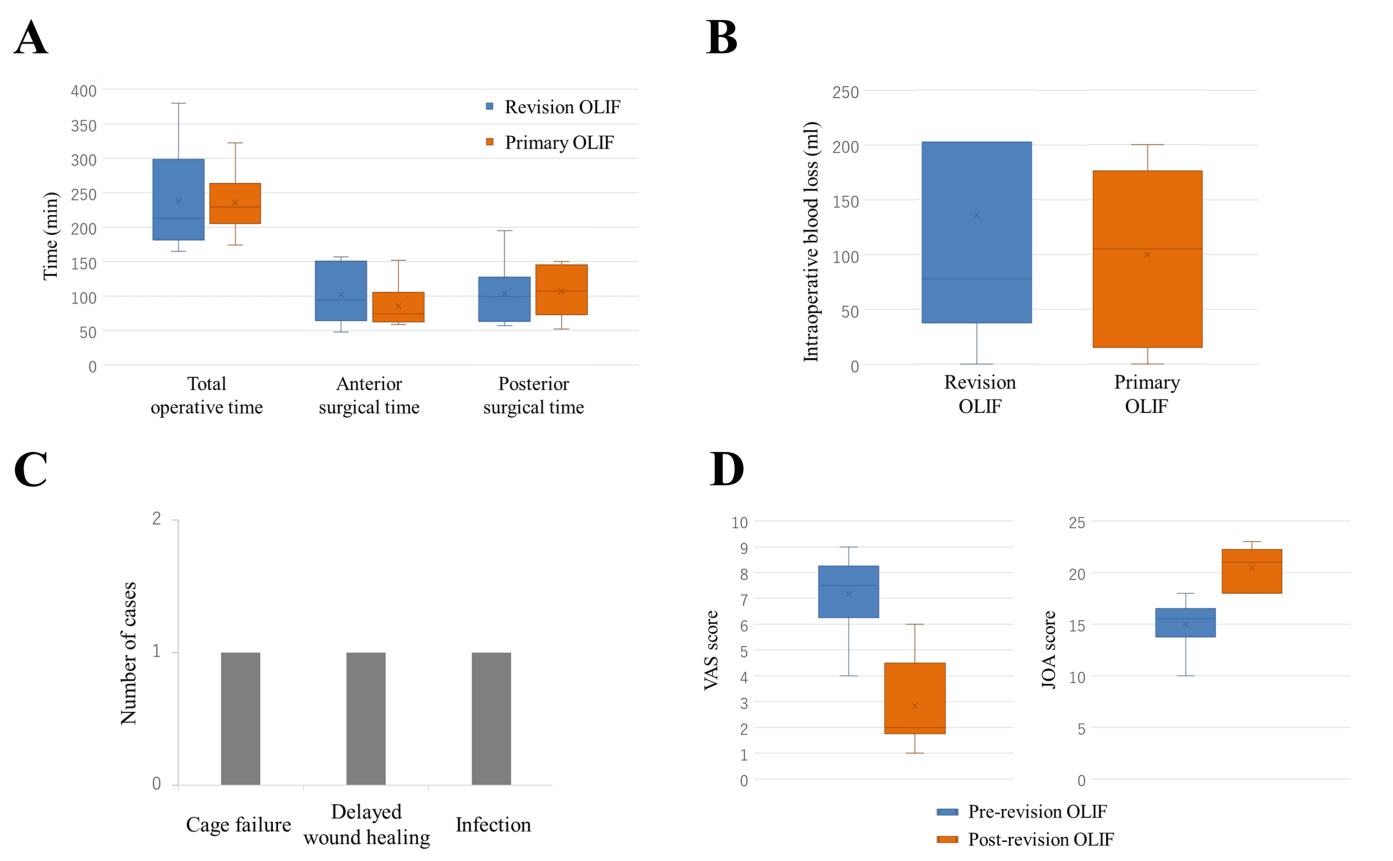
Clinical outcomes. A: Comparison of total, anterior, and posterior operative times between re-OLIF and primary OLIF. Both anterior and posterior surgical times were comparable between the two procedures. B: Comparison of intraoperative blood loss between re-OLIF and primary OLIF. Median blood loss was slightly lower for re-OLIF. C: Summary of perioperative complications observed in re-OLIF cases. Three complications were noted: one cage failure, one delayed wound healing, and one surgical site infection. D: Preoperative and postoperative changes in VAS and JOA scores after re-OLIF. Both scores improved postoperatively. A, B, D: Data are presented as box plots. Boxes represent the interquartile range (IQR), horizontal lines indicate the median, and “×” symbols denote the mean. Whiskers extend to the minimum and maximum values. No outliers are shown. OLIF: oblique lateral interbody fusion; VAS: visual analog scale; JOA: Japanese Orthopaedic Association

All six patients who underwent the attempted re-OLIF successfully completed the procedure, which was performed by four different spine surgeons. The median total operative time for re-OLIF and primary OLIF was 212.5 minutes (IQR, 181.5-298.3) and 229.0 minutes (IQR, 205.5-263.5), respectively. The median anterior surgical time was 94.5 minutes (IQR, 64.5-151.0) for re-OLIF and 74.0 minutes (IQR, 62.8-105.5) for primary OLIF, while the median posterior surgical time was 99.0 minutes (IQR, 63.0-127.5) and 107.5 minutes (IQR, 73.0-145.5), respectively; both anterior and posterior surgical times were comparable between the two procedures (Figure 1A). Median intraoperative blood loss was 77.5 ml (IQR, 37.5-202.5) for re-OLIF and 105.0 ml (IQR, 15.0-176.0) for primary OLIF (Figure 1B). Perioperative complications included one case of cage failure, one case of delayed wound healing, and one case of surgical site infection. The cage failure occurred intraoperatively and was immediately managed by replacing the cage during the same procedure. The delayed wound healing was noted in the early postoperative period and required resuturing. The surgical site infection was a delayed-onset infection that occurred more than one month after surgery and required antibiotic treatment. No peritoneal injury associated with retroperitoneal adhesions was observed (Figure 1C). VAS scores improved from a median of 7.5 (IQR, 6.3-8.3) preoperatively to 2.0 (IQR, 1.8-4.5) postoperatively. JOA scores also improved from a median of 15.5 (IQR, 13.8-16.5) to 21.0 (IQR, 18.0-22.3) after re-OLIF (Figure 1D).

Representative case (no. 1)

**Figure 2:**
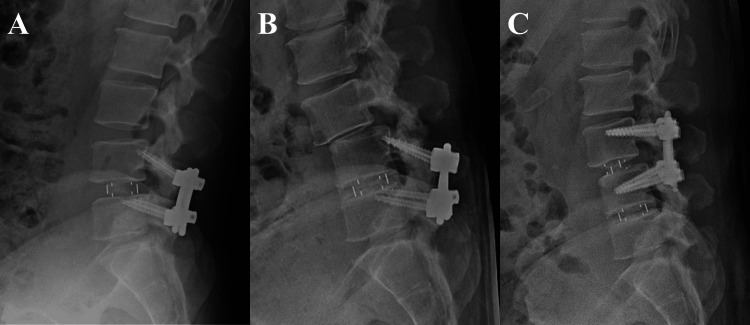
X-ray image of a representative case. A 49-year-old male (age at the time of re-OLIF). A: X-ray image after primary L4/5 OLIF; B: X-ray image taken five years postoperatively showing spondylolisthesis at L3 due to ASD; C: X-ray image after L3/4 re-OLIF with the removal of the L5 screw. OLIF: oblique lateral interbody fusion

A 49-year-old male (age at the time of re-OLIF) underwent primary OLIF at the L4/5 level for anterior spondylolisthesis of L4 (Figure 2A). Five years after primary OLIF, he developed spondylolisthesis of L3 as an ASD (Figure 2B). Six years after the primary OLIF, re-OLIF was performed at L3/4 (Figure 2C). The total operative time was 205 min, the anterior surgical time was 70 min, and the intraoperative blood loss was 70 ml. No perioperative complications were observed. The patient’s VAS score improved from seven to two, and the JOA score improved from 15 to 18 postoperatively. At the time of the latest follow-up, three years had passed since re-OLIF, and no new ASD was observed.

## Discussion

OLIF is a widely used treatment for various lumbar spinal disorders. The usefulness of OLIF as a salvage procedure for ASD following TLIF or PLIF has been well documented in the literature [4-10]. However, whether re-OLIF can be performed as a salvage procedure for ASD after primary OLIF has not been sufficiently explored. As the number of patients with primary OLIF increases and the follow-up periods for these patients increase, the opportunity to encounter ASD after primary OLIF inevitably increases. Therefore, investigating the feasibility of re-OLIF in patients with ASD after primary OLIF is an important clinical question.

In this study, six patients underwent re-OLIF after developing ASD following primary OLIF. Although the sample size was small, re-OLIF was successfully performed in all cases regardless of age, BMI, history of prior abdominal surgery, spinal level at which re-OLIF was attempted, or the interval from the primary surgery. Furthermore, operative time and intraoperative blood loss were comparable between the re-OLIF and primary OLIF groups. These results support the feasibility of re-OLIF, demonstrating that it can be performed without significant complications similar to primary OLIF. Previous reports have shown that the operative time for OLIF varies among institutions. However, the intraoperative blood loss during primary or salvage OLIF after posterior surgery has been consistently reported to be relatively low, typically approximately 100 mL [6-11,19,20]. The intraoperative blood loss after re-OLIF in this study was consistent with previous reports, demonstrating that re-OLIF can be performed with minimal invasiveness.

Regarding complications, one case each of cage failure, delayed wound healing, and infection was observed. These have been frequently reported as typical complications of OLIF surgery [21-23]. In contrast, peritoneal injury, a specific complication associated with the retroperitoneal approach in OLIF, has been reported in 0.8% of cases [23]. Although we anticipated that the risk of peritoneal injury might increase due to adhesions in the surrounding tissues during revision surgery, no such complications were observed in this study.

Although OLIF utilizes a different approach, anterior lumbar interbody fusion (ALIF), which also approaches the vertebral body from the front through the retroperitoneal space via an abdominal incision, is considered difficult in revision surgery because of adhesions around the large vessels [24]. The retroperitoneal space is divided into three regions: the anterior pararenal, perirenal, and posterior pararenal spaces. Unlike ALIF, OLIF uses the posterior pararenal space [25]. The posterior pararenal space does not contain organs and is predominantly composed of fatty tissues. Animal studies have reported that transplantation of epidural fat is useful for preventing epidural adhesion [26] and that preperitoneal fat grafting can prevent intra-abdominal adhesions [27]. Therefore, the posterior pararenal space, which is almost entirely composed of fatty tissue, may be less likely to develop postoperative adhesions. Moreover, OLIF uses finger navigation to develop the posterior pararenal space, thereby avoiding electrosurgery. Electrosurgery, especially when using a coagulation current, has been reported to cause extensive tissue necrosis and an inflammatory response, which increases the risk of adhesions [28]. Therefore, the avoidance of electrosurgery during OLIF may have contributed to the lack of peritoneal injury due to adhesions in this study.

Regarding the VAS and JOA scores after re-OLIF, improvements were observed in all patients postoperatively, and the outcomes were satisfactory. These results are comparable to those previously reported for primary OLIF and OLIF as a salvage procedure after posterior surgery [3,5,6,8-12]. From the perspective of postoperative outcomes, re-OLIF can be considered a useful salvage procedure for ASD following OLIF. However, as no established consensus currently exists regarding the optimal salvage approach for ASD after primary OLIF, further investigation is warranted.

This study has several limitations that should be acknowledged. As a case series, the number of cases was limited, and while re-OLIF was successfully performed without significant complications in all patients included in this study, this result alone cannot definitively establish the procedure as an appropriate salvage strategy for ASD following primary OLIF. In addition, given the retrospective design of this study, the potential for selection bias cannot be excluded. Therefore, further accumulation of cases and additional studies are needed to determine the generalizability and safety of re-OLIF.

Nevertheless, as the number of patients undergoing primary OLIF continues to increase, the incidence of ASD in this patient population is also expected to rise inevitably. In this context, exploring safe and feasible salvage strategies such as re-OLIF is becoming an increasingly important clinical issue.

## Conclusions

In our study, the surgical outcomes of re-OLIF were comparable to those of primary OLIF, with no significant complications or difficulties. As there are no comprehensive reports available on re-OLIF for ASD after primary OLIF, this study provides valuable preliminary insights into its treatment, which is expected to increase in the future. However, given the small sample size, the results should be interpreted with caution. As OLIF becomes more widely adopted, it is anticipated that more ASD cases will arise. Therefore, further accumulation of cases and additional investigations, such as prospective multicenter studies, long-term imaging-based assessments, and comparative analyses with other salvage procedures, are necessary to determine whether re-OLIF can be safely and effectively performed on a larger scale.
